# Non-Sexually Transmitted Infection (STI)-Related Pelvic Inflammatory Disease (PID)

**DOI:** 10.3390/microorganisms13122813

**Published:** 2025-12-10

**Authors:** Eleni Polyzou, Evangelia Ntalaki, Maria Gavatha, Karolina Akinosoglou

**Affiliations:** 1 Faculty of Medicine, University of Patras, 26504 Patras, Greece; polyzou.el@gmail.com (E.P.); gavatha.maria@yahoo.com (M.G.); 2 Department of Internal Medicine and Infectious Diseases, University General Hospital of Patras, 26504 Patras, Greece; 3 Department of Obstetrics and Gynecology, University General Hospital of Patras, 26504 Patras, Greece; evangelia.ntalaki@gmail.com; 4 Division of Infectious Diseases, Department of Internal Medicine, University General Hospital of Patras, 26504 Patras, Greece

**Keywords:** non-sexually transmitted infections, pelvic inflammatory disease, bacterial vaginosis, tubo-ovarian abscess, infertility, sexually transmitted infection, intra uterine device

## Abstract

Pelvic inflammatory disease (PID), although traditionally viewed as a sexually transmitted infection (STI), can also result from non-sexually transmitted microorganisms that display distinct epidemiologic and clinical characteristics. Unlike STI-related PID, these infections are less influenced by sexual behavior, often show a bimodal age distribution, and are linked to bacterial vaginosis (BV)-associated dysbiosis, iatrogenic uterine procedures, postpartum states, or inadequate access to timely screening and care. Non-STI-related PID is usually polymicrobial, predominantly involving BV-associated vaginal, enteric, or urinary commensals that ascend into the upper genital tract, while respiratory tract organisms, mycobacteria, and biofilm-associated pathogens may also play a role. Pathophysiological mechanisms include disruption of the endocervical barrier, mucus degradation, biofilm formation, hematogenous or iatrogenic seeding, and chronic cytokine-mediated inflammation and fibrosis. Clinical manifestations range from asymptomatic/subclinical disease to acute pelvic pain and tubo-ovarian abscess (TOA) and can progress to systemic infection and sepsis. Diagnosing non-STI PID is challenging due to nonspecific symptoms, negative STI tests, and inconclusive imaging findings, while management relies on broad-spectrum antimicrobials with surgery as needed. Given these complexities, this review aims to synthesize current knowledge on non-STI-related PID, clarify key considerations for its diagnosis, management, and prevention, and outline future perspectives to improve clinical outcomes.

## 1. Introduction

Pelvic inflammatory disease (PID) represents a spectrum of infectious and inflammatory conditions affecting the upper female genital tract. It includes endometritis, salpingitis, tubo-ovarian abscess (TOA), and pelvic peritonitis, occurring alone or in combination [1]. Traditionally, PID is considered a sexually transmitted disease (STD) affecting individuals of reproductive age [2]. It is more common among younger women with multiple sexual partners, due to increased prevalence of sexually transmitted pathogens, such as *Chlamydia trachomatis* and *Neisseria gonorrheae* and sexual behaviors that increase exposure risk [3]. A study addressing the global burden of PID has reported that, in 2019, the age-standardized prevalence of PID was 53.19 per 100,000 population, emphasizing its continued impact on reproductive-aged women worldwide [4], whereas other studies similarly reported a high prevalence of PID in young individuals [5].

Surprisingly, recent data reveal that, the percentages of *Chlamydia trachomatis (C. trachomatis)* and *Neisseria gonorrhoeae* (*N. gonorrhoeae)* among women with PID are lower than expected in certain patient groups, while other pathogens are more prevalent [6]. This aligns with reports of pediatric and sexually inactive individuals with PID, in whom *Escherichia coli* and *Streptococcus* species were the predominant pathogens identified [7], highlighting that PID can occur even in the absence of sexual activity [7,8].

Since PID often leads to long-term complications such as chronic pelvic pain, infertility, and ectopic pregnancy [9,10,11], it is essential for clinicians to recognize and evaluate cases early enough, so as to initiate timely management and reduce the risk of adverse outcomes, especially in cases that are not due to sexually transmitted infections (STIs) as might be expected. This review aims to investigate the pathophysiology and underlying mechanisms of non-STI-related PID, the differences in clinical presentation compared to STI-related cases, the associated diagnostic and management challenges, and potential avenues for future research and improved clinical strategies.

## 2. Epidemiology

PID is strongly influenced by behavioral risk factors, showing a clear association with non-STI-related PID. Vaginal douching, for example, increases risk by disrupting protective lactobacilli in the vaginal microbiota [12]. In contrast to STI-associated PID, sexual behaviors demonstrate only a weak correlation with non-STI-related cases, a distinction of importance for preventive strategies. Emerging evidence also suggests an association between gastrointestinal health and PID, with higher incidence observed among women with inflammatory bowel disease (IBD) or those with frequent antibiotic use [13]. Major risk factors also include having multiple sexual partners, not using barrier contraception, and being under 25 years of age. The chance of recurrence is increasing following previous PID incidents, due to the preceding tubal damage, maintaining a cycle of ongoing inflammation and infertility [14,15,16,17].

Non-STI PID accounts for approximately 15% of total PID cases, although this proportion may vary across world regions [18]. In high-income countries (Gross National Income per capita > $13,935), studies indicate that 55–62% of PID cases are not attributable to commonly prevalent sexually transmitted pathogens, including *C. trachomatis*, *N. gonorrhoeae*, or *M. genitalium* (UK: 61.8%; Australia: 55%), highlighting a possibly substantial burden of non-STI PID [19,20,21]. In contrast, low-income countries (Gross National Income per capita ≤ $1135) face limited access to diagnostic testing for STIs, likely resulting in underdiagnosis of sexually transmitted PID and a higher observed proportion of non-STI PID [21,22]. Additionally, unsafe gynecological procedures in low-income countries, including an estimated 21.6 million unsafe abortions annually, contribute significantly to PID in these regions, causing approximately 68,000 maternal deaths, 5 million hospital admissions, and 1.7 million cases of secondary infertility each year. These data underscore clear epidemiological disparities in non-STI PID between high- and low-income countries [23].

Geographically, non-STI-related PID prevalence has also been related to bacterial vaginosis (BV) prevalence (higher in Black and Hispanic women in the U.S.) and limited healthcare access in regions with poor STI screening. Approximately 45% of women with BV show subclinical endometritis [14].

Epidemiological patterns specific to age demonstrate significant differences between STI and non-STI-related PID. STI-related PID mainly impacts sexually active women between 15–24 years, whereas non-STI-related PID shows a bimodal age distribution [16,17]. Girls under 15 may sometimes experience PID due to hematogenous spread from gastrointestinal infections such as appendicitis [24], while women aged ≥35 years are more likely to develop PID due to BV, intrauterine device (IUD) use, and postpartum infections [12,16,17]. Ιt is noted that, PID cases requiring hospitalization are more likely to involve enteric bacteria (e.g., *E. coli*, *Bacteroides*, etc.) and abscess formation, suggesting non-STI-related etiologies drive severe disease [25,26].

Temporal patterns, however, indicate changing epidemiology. Analysis of PID cases in the U.S. from 2006 to 2016 revealed that, although STI-related PID dropped significantly (6.5% per year in younger women), non-STI PID showed a slower decline (1.4% per year in women aged 30 and older). The shift toward a higher proportion of non-STI PID intensified after 2012, coinciding with increased IUD usage and stable BV prevalence, suggesting that non-STI-related PID may represent a growing proportion of total cases in developed countries [16].

Certain medical procedures, particularly IUD insertion, are associated with a transient increase in PID risk, most notably within the first 21 days post-placement, likely due to the introduction of bacteria during the procedure. An important distinction emerges in etiology: while STIs account for the majority of PID cases among non-IUD users, IUD-associated PID more frequently occurs in women without STIs, suggesting alternative mechanisms, such as ascending vaginal flora or bacteria introduced at the time of insertion. Despite this short-term risk, the overall long-term risk of PID among IUD users remains low. These findings underscore the importance of STI screening prior to IUD placement to avoid introducing an additional risk factor for PID [14,27]. Hence; findings are consistent with current guidelines recommending STI screening prior to IUD insertion to mitigate the risk of PID, yet they also challenge the assumption that IUD-associated PID occurs exclusively in the presence of STIs [28]. It is important to consider that non-STI pathogens, such as bacteria associated with BV, may contribute to PID in IUD users, particularly among those who test negative for common STIs [29].

Surgical interventions, such as hysteroscopy and curettage, may disrupt the cervical mucosal barrier, facilitating the ascent of vaginal flora and subsequent infection. Procedures that breach the endocervical canal, including hysterosalpingography (HSG), sonohysterography, IUD insertion, and endometrial sampling, are classified as clean-contaminated. Despite the absence of routine antimicrobial prophylaxis, the overall risk of infection following these interventions remains low. However, specific circumstances—such as a prior history of PID or abnormal tubal anatomy detected during HSG or laparoscopic chromopertubation—are associated with an elevated risk of postoperative PID or endometritis, warranting perioperative precautions and targeted antimicrobial prophylaxis. Antimicrobial regimens should be selected with consideration for the polymicrobial etiology of these infections. Although uncommon, PID following HSG occurs in approximately 1.4–3.4% of cases and may represent a serious complication [30,31]. Patients with dilated fallopian tubes identified during HSG demonstrate a significantly increased incidence of post-procedural PID, reported at approximately 11% [32].

## 3. Microbiology

Three primary categories of pathogens linked to PID exist, which can overlap: (1) sexually transmitted pathogens (such as *N. gonorrhoeae*, *C. trachomatis*, *Mycoplasma genitalium* (*M. genitalium*), *Trichomonas vaginalis*), (2) bacteria associated with BV (like BVAB3, *Prevotella bivia*, *Atopobium vaginae*, *Leptotrichia/Sneathia* spp.), and (3) bacteria from the gastrointestinal (GI) or respiratory tract (for instance, anaerobes, as well as facultative and aerobic bacteria such as *Haemophilus influenzae (H. influenzae)*, *E. coli*, *Bacteroides* [14,15].

Microbiological research indicates that 25–30% of PID cases with non-STI pathogens can be associated with anaerobic bacteria of BV, as well as genital *Mycoplasmas* and *Ureaplasmas*, especially in women with vaginal dysbiosis with decreased *Lactobacillus* predominance [33,34]. Anaerobes associated with BV, such as *Prevotella*, *Atopobium* are the most prevalent, found in 25–62% of instances. Moreover, pathogens linked to BV, including respiratory pathogens like *H. influenzae*, *S. pneumoniae*, and *S. aureus* have been associated with acute PID [12,35].

Enteric organisms such as *E. coli* and *Bacteroides fragilis* represent 18–22% of incidents, especially after gynecological procedures. *E. coli*, *Enterococcus faecalis*, and *Bacteroides* species are important in around 20–30% of PID instances, especially in cases that are chronic, recurrent, or post-procedural. These organisms probably access the upper genital tract via ascending transmission from the rectal/vaginal region, hematogenous spread (particularly postpartum), or iatrogenic introduction during gynecological procedures [36]. Genital mycoplasmas (*M. hominis)*, *Ureaplasma urealyticum* are found in 6–12% of instances, whereas new pathogens such as *Fusobacterium* are involved in chronic PID [15,37].

Other non-STI pathogens show specific epidemiologic patterns: *Fusobacterium nucleatum* is identified in 28–34% of chronic PID cases and in 42% of STI-negative women, while *Actinomyces israelii* is strongly linked to prolonged IUD use (>2 years) and associated with granulomatous pelvic disease. In low-resource or endemic regions, genital tuberculosis remains a significant but often overlooked cause, typically affecting women under 40 and disproportionately impacting non-Hispanic Black patients and those with alcohol abuse behaviors [38,39]. Female genital tract tuberculosis (FGTB) due to *Mycobacterium Tuberculosis* (*M. tuberculosis*) is a form of genitourinary TB, usually secondary to pulmonary disease via hematogenous spread, accounting for 5% of all pelvic female infections [40]. FGTB should always be considered in low- and middle-income or other high-TB-burden settings when evaluating women with infertility, chronic pelvic pain, abnormal uterine bleeding, or adnexal masses [41].

Notably, available data from cohort studies of non-sexually active adolescent females with PID/TOA reveal a predominance of enteric and urinary pathogens, most commonly *E. coli*, *Bacteroides* spp. and *Streptococcus* spp., with *N. gonorrheae* and *chlamydia* typically not detected [42].

Despite increasing recognition of non-STI pathogens in PID, data on their true prevalence remain fragmented, and no systematic review has comprehensively synthesized their epidemiology and clinical patterns. For this purpose, a literature search was conducted by three independent reviewers over a 30-year period (1995–2025) to identify cases or case series of individuals with PID or TOAs associated with non-sexually transmitted organisms. The search was performed in PubMed using the following MeSH terms: pelvic inflammatory disease, tubo-ovarian abscess, endometritis, non-sexually transmitted infections, *Actinomyces*, *Mycobacterium tuberculosis*, non-sexually active, oophoritis, salpingitis, *Escherichia coli*, anaerobes. Because our review focused specifically on individual case reports and case series, PubMed was chosen as the optimal source, as it contains the largest curated collection of peer-reviewed medical case reports globally. Inclusion criteria required that cases presented with clinical symptoms and a diagnosis consistent with non-STI-related PID, and that the causative microorganism was identified in each report. The presence of an abstract was also mandatory for eligibility. Exclusion criteria included PID associated with sexually transmitted pathogens, as well as reports without an abstract or without identification of a specific causative microorganism. The identification, screening, and inclusion of reports were conducted according to the PRISMA framework, as depicted in the Supplementary Materials, Figure S1. All records and full-text reports were screened by three reviewers working independently, with disagreements resolved by consensus. No automation tools were used in any stage of the screening or selection process. Duplicate records were identified and removed using manual review. Articles that were not published in English or did not involve human subjects were excluded from screening to ensure relevance and applicability to clinical practice, whereas an additional 3814 articles did not meet the specific criteria of our search, resulting in 510 reports that were assessed for eligibility. From the 510 reports, studies were further excluded for the following reasons: no abstract available (n = 111), unrelated title (n = 110), no microorganism identified (n = 131), among others, resulting in the final set of 159 reports, case series and cohort studies included in the review. Given the descriptive nature of case reports and the heterogeneity of reported data, meta-analysis was not feasible, and findings were synthesized narratively.

Table 1 summarizes microbiology of non-STI-related PID cases identified in literature since 1995 (Methodology in Supplementary Materials, Figure S1).

**Figure 1:**
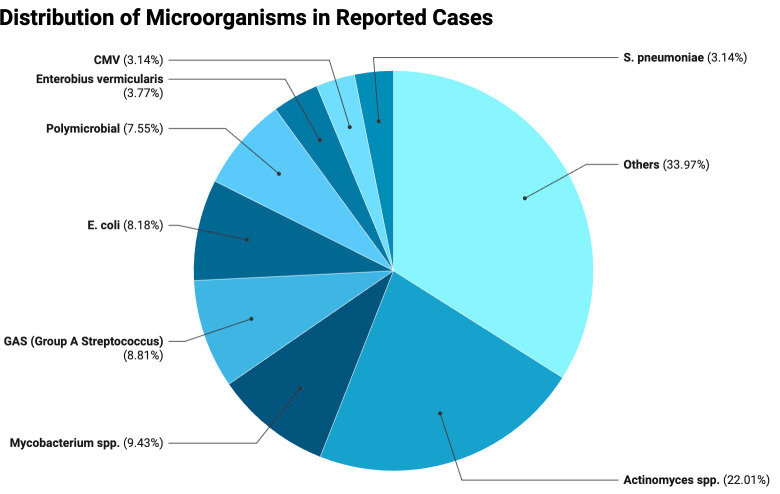
Distributions of microorganisms in reported cases.

When evaluating the proportions as shown in the pie chart (Figure 1), *Actinomyces* spp. represented the largest share of cases (22.01%), followed by *Mycobacterium* spp. (9.43%), GAS (8.81%), *E. coli* (8.18%), and polymicrobial infections (7.55%). Less frequent isolates included *Enterobius vermicularis* (3.77%), CMV, and S. *pneumoniae* (each 3.16%), while the remaining 33.96% comprised various microorganisms in smaller proportions.

When interpreting these results, certain limitations should be taken into account. The search was restricted to PubMed, which may have led to exclusion of relevant reports indexed in other databases. The evidence base consists predominantly of case reports and small case series, making the findings susceptible to publication bias and selective reporting. Because the presence of an abstract was required for eligibility, some potentially relevant non-indexed reports may have been excluded. Moreover, case reports do not provide denominators, preventing any estimation of true prevalence or incidence. Finally, limiting inclusion to English-language publications may have introduced language bias and underrepresented data from regions where non-STI etiologies, such as tuberculosis, are more common.

Thus, consistent with the findings of our review, *Actinomyces* species represent a notable non-STI pathogen associated predominantly with chronic PID. Their prevalence is particularly higher among IUD users, with reported rates reaching approximately 2–4% in screened populations and occurring more frequently after prolonged device use [197]. Colonization rates may be even higher, as up to 7% of IUD users can have Actinomyces-like organisms detected on cervical cytology, although this finding has limited predictive value for true infection, making it difficult to determine the real prevalence of clinically significant disease [198]. However, the notably higher proportion of Actinomyces observed in our review should be interpreted with caution, as rare pathogens are often overrepresented in case reports, which tend to emphasize unusual or atypical presentations. In the same line, although FGTB accounts for a substantial proportion of PID cases in endemic regions, often presenting with infertility [199], the proportion observed in our review is higher because it reflects only the distribution among non-STI-related PID cases rather than the prevalence in the general PID population. Further well-designed cohort studies focusing exclusively on non-STI PID are needed to more accurately characterize its epidemiology and clinical burden.

## 4. Pathophysiology

**Figure 2:**
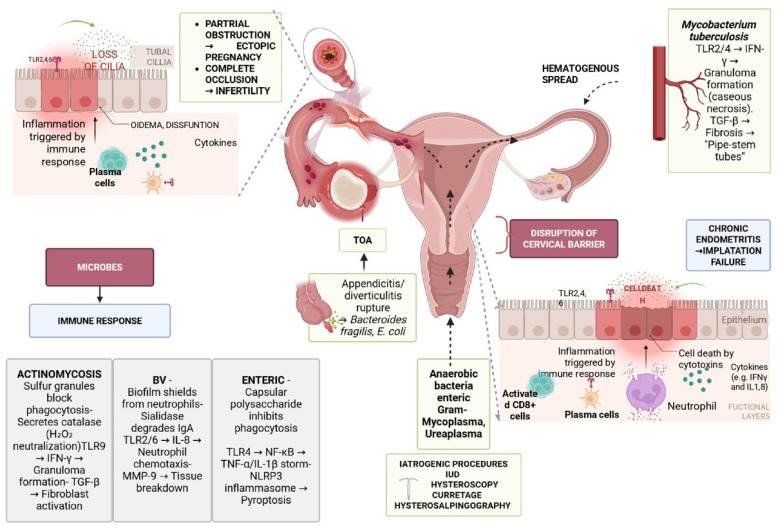
Pathophysiology of PID (TOA: tubo-ovarian abscess; TLR: Toll-like receptor, IFN-γ; interferon gamma; TGF-β: transforming growth factor beta; IL-8: interleukin-8; IUD: intrauterine device; *E. coli*: *Escherichia coli*; MMP-9: matrix metalloproteinase-9; IL-1: interleukin-1; IgA: immunoglobulin A; NF-κB: nuclear factor kappa-light-chain-enhancer of activated B cells).

PID results from disruption of the endocervical barrier, normally protecting the sterile upper genital tract from the polymicrobial vaginal environment [14,15]. Classically, *Chlamydia trachomatis* and *Neisseria gonorrhoeae* ascend during menses, damaging the tubal epithelium, destroying cilia, and triggering edema and inflammatory infiltration, which lead to obstruction, infertility, or ectopic pregnancy [200,201,202]. However, non-STI pathogens are increasingly recognized as key drivers of PID, often acting synergistically with or independently of STIs [14,15]. BV-associated anaerobes such as *Gardnerella vaginalis*, *Prevotella*, and *Atopobium* degrade cervical mucus, weaken mucosal defenses, and fuel cytokine-mediated fibrosis [203,204,205], while *M. genitalium*, *Ureaplasma*, and *M. hominis* exploit immune evasion and biofilm formation to sustain chronic inflammation [34,200,201,202,206]. Enteric bacteria including *E. coli*, *Bacteroides*, and *Enterococcus faecalis* reach the adnexa through ruptured appendicitis, diverticulitis, or intra-abdominal abscesses, frequently causing TOAs [207,208]. Other non-STI agents include *Fusobacterium nucleatum*, which invades via E-cadherin, suppresses immune clearance, and forms resistant biofilms [37,209,210], and *Actinomyces israelii*, particularly in long-term IUD users, which produces granulomatous inflammation with sulfur granules, fibrosis, and fistulae [71,72,211,212]. In endemic settings, *M. tuberculosis* disseminates hematogenously to the pelvis, causing granulomatous salpingo-oophoritis and peritonitis. Unlike STI-driven PID, which typically presents acutely with ascending infection, non-STI PID is often polymicrobial, insidious, and characterized by biofilm formation, host–pathogen synergy, and chronic tissue remodeling [213]. Collectively, these mechanisms explain why PID can occur in women without classical STIs and underscore the need to distinguish STI from non-STI-associated disease. Figure 2 summarizes main pathophysiological mechanisms implicated in PID with emphasis in non-STI PID.

## 5. Clinical Manifestations

PID covers a broad range of clinical manifestations. A substantial proportion of women diagnosed with PID exhibit no clinical signs of genital tract infection, while others become aware of the condition only after a diagnosis of tubal factor infertility. Acute symptomatic PID presents with a broad spectrum of clinical manifestations, ranging from mild, nonspecific pelvic discomfort to severe pain associated with TOAs [214]. The progression of the presentation is generally rapid over a few days, although a slower presentation may occur over weeks to months.

STI-related PID is characterized by acute symptomatic onset, typically presenting as the sudden development of lower abdominal or pelvic pain. Symptoms rarely persist beyond two weeks and are commonly accompanied by pelvic organ tenderness and clinical evidence of genital tract inflammation [1,215,216,217]. Most females with PID experience mild to moderate disease, with only a small percentage developing peritonitis or pelvic abscess, typically presenting with more intense pain, increased tenderness on examination, dyspareunia and systemic symptoms [14,218]. Additional nonspecific symptoms may include increased urinary frequency and abnormal vaginal discharge. When comparing chlamydia-associated PID with *M.genitalium*–associated PID, patients generally reported similar clinical manifestations; however, cases attributed to *M.genitalium* demonstrated lower rates of postcoital bleeding but greater lower abdominal tenderness [202].

Of particular note, it is estimated that a large proportion of cases are associated with chronic PID, a slowly progressive form of the condition typically characterized by low-grade fever, weight loss, and persistent abdominal discomfort, and reported predominantly in cases with non-sexually transmitted etiologies [219]. Pelvic actinomycosis manifests as chronic pelvic pain, weight loss, and mass-like lesions that can mimic malignancy or tuberculosis [46,48,51,65]. Similarly, *Candida albicans*–associated TOAs can develop months to years after IUD placement. Tuberculous and polymicrobial endometritis typically cause secondary amenorrhea, infertility, abnormal bleeding, pelvic pain, and fever [105,115,132,133,138,180].

Certain rare presentations may be misleading or severe. Xanthogranulomatous inflammation can simulate ovarian cancer, presenting with atypical abdominal pain and a pelvic mass. Severe systemic presentations include sepsis or toxic shock syndrome due to *Streptococcus pyogenes* or *Fusobacterium*, and in immunocompromised or postmenopausal women, *E. coli* or CMV-related abscesses may occur [76,84,86,91,92,94,103,108,110,125,130,131,180,181].

Postpartum infections constitute another subgroup. They often present with fever, abdominal pain, or sepsis, commonly caused by *Eggerthella lenta*, GAS, or CMV, while cesarean-associated cases may involve uterovesical abscesses [89,90,111,122,143,150,182].

## 6. Diagnosis

Diagnosis of PID is challenging due to the wide variation in clinical presentation, many women have mild, nonspecific, or no symptoms and because it is primarily a clinical diagnosis. Moreover, in cases of non-STI-related PID, the absence of laboratory-confirmed cervical infection with *N. gonorrhoeae* or *C. trachomatis* further complicates the diagnostic process [1]. A differential diagnosis for PID should include conditions that can present with similar lower abdominal or pelvic pain and associated symptoms. These include ectopic pregnancy, ruptured ovarian cyst, adnexal torsion, and endometriosis. Urinary tract infections, such as cystitis or pyelonephritis, as well as gastrointestinal conditions like appendicitis, diverticulitis, or irritable bowel syndrome, should also be considered. Additionally, traumatic injury to the pelvic region may mimic the clinical presentation of PID and should be evaluated [220].

### 6.1. Medical History and Physical Examination

The assessment of medical history in cases of PID frequently emphasizes sexually transmitted risk factors, such as age below 25 years, multiple sexual partners, a partner with a known STI or related symptoms, early initiation of sexual activity, previous STIs, inconsistent use of barrier contraception, and the presence of bacterial vaginosis. However, these parameters may be of limited value in identifying non-STI-related PID [220].

In addition, other risk factors have been reported. The insertion of an IUD, particularly within the first three weeks post-insertion, has been associated with an increased risk of PID [221]. A detailed surgical history is also essential, as actinomycosis unrelated to IUD use is almost invariably linked to prior surgical procedures [222]. Furthermore, previous bowel surgery has been identified as a risk factor for PID in individuals without sexual activity [223].

According to the Centers for Disease Control and Prevention (CDC), the minimum clinical diagnostic criteria for PID include cervical motion tenderness, uterine tenderness, or adnexal tenderness. These criteria primarily apply to sexually active women at risk of STIs [1]. Consequently, their specificity is reduced in cases of non-STI-related PID. Patients with non-STI-related PID commonly present with lower abdominal pain, fever, and gastrointestinal disturbances, and may report a history of urinary tract infections, congenital anomalies, or appendicitis [7]. Additional symptoms may include bilateral lower abdominal pain, dyspareunia, abnormal uterine bleeding, menorrhagia, and abnormal vaginal or cervical discharge, often associated with cervicitis, endometritis, or BV [215].

### 6.2. Diagnostic Workup

No single physical or laboratory finding demonstrates both high sensitivity and specificity for the accurate diagnosis of acute PID. Although combining multiple diagnostic criteria may improve sensitivity—thereby increasing the detection of true positive cases—or specificity—enhancing the exclusion of false positives—such improvements are typically achieved at the expense of the other parameter. [1].

Serum inflammatory markers such as erythrocyte sedimentation rate (ESR), C-reactive protein (CRP), and white blood cell count (WBC) have limited diagnostic specificity and are often normal in mild or moderate cases of PID; thus, they serve only a supportive role in diagnosis [215]. All patients should undergo nucleic acid amplification tests (NAATs) [224], including real-time PCR from endocervical swabs to detect *C. trachomatis*, *N. gonorrhoeae and M. genitalium*. Additionally, serological tests for *T.pallidum*, HIV, as well as a pregnancy test to rule out ectopic pregnancy are recommended [215].

Endometrial cultures may provide useful adjunctive information in patients with clinically suspected acute disease, while laparoscopic evaluation with intra-abdominal bacterial cultures can be valuable in severe cases, both for diagnostic confirmation and for guiding targeted antimicrobial therapy [225,36].

Other laboratory tests, including tumor markers such as Cancer Antigen 125 (CA-125), may also contribute to assessing the extent and potential complications of the disease [226,227].

### 6.3. Microscopy

Microscopic examination of vaginal and endocervical secretions may reveal WBCs or mucopurulent cervical discharge, findings that can suggest PID but lack diagnostic specificity. In contrast, the absence of pus cells or WBCs on a saline wet mount has a high negative predictive value (approximately 95%), making a diagnosis of PID unlikely [1,215].

### 6.4. Imaging

#### 6.4.1. Ultrasound

Pelvic ultrasonography, particularly transvaginal ultrasound, is the preferred initial imaging modality for evaluating pelvic pain in nonpregnant women [228,229]. Ultrasound can identify inflammatory changes in the fallopian tubes, with color Doppler imaging demonstrating features such as edema, wall thickening, and increased vascularity. In advanced stages of PID, the inflammation may extend to the ovaries, manifesting as complex adnexal masses with indistinct margins and fluid-filled areas, which can occasionally mimic ovarian malignancy [228].

#### 6.4.2. Computed Tomography (CT)

When ultrasound findings are inconclusive or the procedure cannot be performed due to patient discomfort, CT serves as an important modality [230]. CT scan can identify lesions associated with PID, such as cervicitis, endometritis, acute salpingitis, oophoritis, pyosalpinx, hydrosalpinx, TOA, and pyometra, as well as complications of PID, including tubal damage leading to infertility or ectopic pregnancy, peritonitis from ruptured abscesses, peritoneal adhesions causing bowel obstruction or hydroureteronephrosis. CT is also effective in evaluating conditions that may mimic PID, such as endometriosis, adnexal torsion, ruptured hemorrhagic ovarian cyst, adnexal neoplasms, appendicitis, and diverticulitis [231].

#### 6.4.3. Magnetic Resonance Imaging (MRI)

MRI can aid in narrowing down differential diagnoses and establishing an accurate diagnosis. MRI offers high diagnostic accuracy in detecting PID and TOAs [229]. MRI can identify adnexal lesions that appear T1 hypointense and T2 hyperintense, often demonstrating diffusion restriction, thickened walls, internal septations, and strong post-contrast enhancement, features highly suggestive of TOAs [232].

### 6.5. Transcervical Endometrial Biopsy

Endometrial biopsy can aid in the diagnosis of PID by identifying histologic signs of endometritis, specifically the presence of neutrophils and plasma cells in the endometrium [200]. It is a minimally invasive procedure that offers an alternative to laparoscopy, particularly when there is diagnostic uncertainty. A negative result has a high negative predictive value for ruling out upper genital tract infection. However, its clinical utility is limited by the need for specialized pathology expertise, delayed turnaround time, and restricted availability [233].

### 6.6. Laparoscopy

Laparoscopy plays a crucial role in the diagnosis of PID, especially in cases where there is no improvement within 72 h of antibiotic therapy or when imaging shows thickened fallopian tubes, intra-abdominal fluid, or TOAs. It allows for direct visualization of the pelvic organs, helping to confirm the diagnosis, rule out other conditions like appendicitis or endometriosis, and obtain bacterial swabs for microbiological culture [234]. Although considered the gold standard for diagnosing PID, the need for general anesthesia, hospitalization, and surgical expertise significantly limits the routine use of diagnostic laparoscopy [233].

### 6.7. Biomarkers

Traditionally, CA-125, a glycoprotein produced by coelomic epithelial cells and widely used as a tumor marker, has also been investigated in benign conditions [235]. Elevated levels have been associated with peritoneal involvement in diseases such as PID, endometriosis, uterine fibroids, adenomyosis, and various non-malignant serous effusions, supporting its potential role as a prognostic biomarker and in monitoring treatment response in non-malignant conditions [235].

Other biomarkers, commonly used in the evaluation of infectious diseases [236] such as procalcitonin (PCT) and neutrophil-to-lymphocyte ratio (NLR), have been evaluated in the diagnosis and management of PID and TOA [237]. Elevated levels of these markers have been associated with an increased likelihood of surgical intervention [237]. Similarly, immature granulocytes (IGs) can serve have been proposed as markers of disease severity, as elevated levels have been shown to correlate with more severe clinical presentations of PID [238].

## 7. Management and Treatment

Current international guidelines do not prioritize pathogen-directed therapy for PID. Although it is recognized that the proportion of PID attributable to classic STIs has decreased and a wider range of microorganisms is now implicated, existing CDC and European guidelines continue to recommend empiric, broad-spectrum regimens rather than tailored antimicrobial approaches based on the causative pathogen [1,215]. Moreover, the low prognostic value of screening tests to rule out upper-genital-tract infection has made tailored, pathogen-directed treatment difficult, as negative results do not reliably exclude involvement of key organisms [1]. Other international guidelines, including those from Australia and Canada, similarly focus on STI-related causes of PID and do not provide pathogen-specific recommendations for non-STI etiologies [239,240]. Table 2 summarizes treatment regimens specific to the pathogens identified in the reported cases above, alongside recommended empiric therapy and alternative regimens for patients with allergies or other contraindications. These pathogen-directed treatments are based on existing guidelines for abdominal or site-specific infections caused by the same organisms and represent therapeutic options that could be applied to PID when these pathogens are implicated.

### 7.1. Empiric Treatment

Given the risk of long-term complications, such as infertility and ectopic pregnancy, both diagnosis and treatment should be initiated promptly upon clinical suspicion of PID. Early intervention is essential to reduce the risk of irreversible reproductive damage [1]. Several factors may influence therapeutic decision making, including local antimicrobial resistance patterns, regional epidemiology, treatment costs, patient preferences and adherence, and disease severity [215]. The choice between oral and parenteral regimen should be guided by certain criteria such as the severity of the illness, tolerance of oral medications, and the presence of complicating factors, like pregnancy or suspected abscess or other surgical emergencies. For mild to moderate cases, oral and parenteral regimens appear to be equally effective. Hospitalization is indicated for patients with severe clinical presentations, inability to adhere to outpatient therapy, or lack of response to initial oral therapy [1,215,221,241]. Parenteral regimens should be preferred in pregnant patients or in cases of diagnostic uncertainty, ensuring optimal management and minimizing complications. Importantly, adolescents should be managed using the same clinical criteria as adults, as age alone does not necessitate inpatient care [1,215,221,241]. Overall, the antibiotics used for treating PID have high efficacy with favorable safety profiles, most of which are well-tolerated and associated with only mild, manageable side effects [242].

In the management of non-STI-related PID, the therapeutic approach must address two pathogen spectra. In addition to sexually transmitted pathogens such as *N. gonorrhoeae*, *C. trachomatis*, *M. genitalium*, and *Trichomonas vaginalis*, coverage should extend to BV–associated organisms (e.g., *Atopobium vaginae*, *Sneathia*, *Megasphaera*), as well as enteric and respiratory tract-associated bacteria like *Bacteroides*, *E. coli*, *Streptococcus* species, and *H. influenzae* [15].

Nonetheless, all treatment regimens for PID should provide coverage for *N. gonorrhoeae* and *C. trachomatis* due to the fact that negative endocervical screening does not reliably exclude upper genital tract infection [1,243]. Recommended regimens are presented in Table 2.

#### 7.1.1. Hospitalized Patients

For hospitalized patients, parenteral therapy should include ceftriaxone in combination with doxycycline and metronidazole. Cefoxitin or cefotetan in combination with doxycycline and metronidazole can achieve equivalent therapeutic efficacy [1]. Recent updates in treatment recommendations indicate that metronidazole should be included as a mandatory component of empiric therapy for PID, to ensure adequate coverage of anaerobic organisms [244].

Alternative regimens include combinations such as clindamycin plus gentamicin, or ampicillin–sulbactam combined with doxycycline, both of which provide effective coverage against *N. gonorrhoeae*, *C. trachomatis*, and anaerobic bacteria. Other options include azithromycin for a 7-day course combined with metronidazole for 14 days, ampicillin plus clindamycin with gentamicin, levofloxacin plus metronidazole, or imipenem with cilastatin [1]. These regimens may serve as comparably effective alternatives when first-line therapies are contraindicated or unavailable. Due to the discomfort often associated with intravenous infusion, doxycycline is preferably administered orally when tolerated, as its oral formulation offers bioavailability comparable to that of the intravenous form [1].

Transition to oral therapy should be considered after 24 to 48 h of sustained clinical improvement. The recommended oral regimens consist of doxycycline combined with metronidazole to complete a total 14-day course of therapy. For patients unable to tolerate doxycycline, azithromycin may be used as an alternative, while clindamycin can substitute for metronidazole to maintain anaerobic coverage [1]. Despite less robust evidence, European guidelines recommend a 14-day regimen of IV ofloxacin plus IV metronidazole as an alternative treatment option for PID [215]. On the contrary, the CDC does not recommend quinolones for routine PID treatment due to the increasing prevalence of quinolone-resistant *N. gonorrhoeae.* However, in select cases—such as patients with cephalosporin allergy or low risk of gonorrhea—close follow-up-quinolone-based regimens may be considered. In these circumstances, levofloxacin or moxifloxacin combined with metronidazole for 14 days may be used [1].

#### 7.1.2. Non-Hospitalized Patients

Outpatient therapy is appropriate for females with mild to moderate pelvic in PID who can tolerate oral medications and are likely to adhere to the treatment regimen. The recommended approach includes a single intramuscular (IM) dose of a long-acting cephalosporin, preferably ceftriaxone, combined with doxycycline for 14 days and metronidazole for 14 days. Alternative cephalosporins that may be used include cefoxitin, cefotaxime, or ceftizoxime. For patients who cannot tolerate doxycycline, azithromycin can be substituted for a complete 14-day course, although it should still be combined with a cephalosporin and metronidazole [1].

Emerging data on alternative treatments, including morinidazole in combination with levofloxacin, suggest promising efficacy and safety profiles, particularly in the context of increasing resistance to standard agents such as metronidazole [245].

### 7.2. Enteric Pathogens

In certain cases, the formation of a TOA arises from localized extension of infection secondary to uncontrolled inflammatory bowel disease, appendicitis, adnexal surgery, or, less commonly, hematogenous spread from distant organs [246,247]. The microbiology profile of TOAs tends to be polymicrobial, involving organisms such as *E. coli*, aerobic *streptococci*, *B.fragilis*, *Prevotella*, and other anaerobes. Thus; antimicrobial regimens must provide adequate coverage for this broad spectrum of organisms [248]. Although management generally adheres to CDC-recommended guidelines, there is rising concern regarding potential resistance of bowel flora to agents such as cefotetan and cefoxitin [249]. Given that bowel flora are often involved in TOA pathogenesis and the classification of TOA as a serious intra-abdominal infection, broader-spectrum regimens such as ertapenem [250], meropenem, or piperacillin-tazobactam may represent more effective therapeutic options [251,252]. Empiric therapy for *Enterobacterales* should be stratified by illness severity, patient specific factors (allergies, immunocompromise) [253] and *Pseudomonas* risk factors which include recent hospitalization, devices, recent IV antibiotics, immunosuppression [254,255]. In stable, immunocompetent patients without healthcare exposures, a broad-spectrum single agent (e.g., a third- or fourth-generation cephalosporin) is appropriate. With healthcare exposures or immunosuppression and in cases of sepsis/septic shock a single antipseudomonal β-lactam (e.g., cefepime/ceftazidime, piperacillin–tazobactam, or a carbapenem) is preferred. For severe β-lactam allergy, aztreonam or a fluoroquinolone may serve as equal alternatives. Severe sepsis/septic shock warrants empiric combination coverage for Gram-negatives (β-lactam plus aminoglycoside rather than fluoroquinolone) [256]. Carbapenem therapy with meropenem, imipenem–cilastatin, or ertapenem is favored for extended spectrum b lactamases (ESBL)-E infections [253]. Ceftolozane–tazobactam, a newer antipseudomonal β-lactam/β-lactamase inhibitor has excellent activity against multidrug-resistant *P. aeruginosa*, including many strains resistant to piperacillin–tazobactam, ceftazidime, or cefepime though it does not overcome carbapenemase producers [253]. When AmpC β-lactamase production is suspected in organisms with a moderate risk profile, cefepime is an appropriate treatment option [253]. For Class A (KPC) β-lactamases, preferred regimens include meropenem–vaborbactam, ceftazidime–avibactam, or imipenem–cilastatin–relebactam, with cefiderocol as an alternative. For Class B (MBLs such as NDM/VIM/IMP), the preferred approach is ceftazidime–avibactam combined with aztreonam, or cefiderocol as monotherapy. For Class D (OXA-48–like) producers, ceftazidime–avibactam is preferred, with cefiderocol as an alternative [253].

### 7.3. Anaerobic Pathogen Associated Infections

In case of anaerobic infections, metronidazole is a first-line option. Clindamycin covers many anaerobes but rising resistance in B. fragilis makes it less reliable than metronidazole, β-lactam/β-lactamase inhibitor (BL/BLI) combinations, or carbapenems for empiric therapy. BL/BLI regimens (e.g., amoxicillin–clavulanate, ampicillin–sulbactam, ticarcillin–clavulanate, piperacillin–tazobactam) also provide broad anaerobic coverage. Among cephalosporins, second-generation agents (cefoxitin, cefotetan, cefmetazole) are more active against *B. fragilis* but, due to increasing resistance, are not recommended for empiric treatment and are used primarily for surgical prophylaxis. Carbapenems (imipenem, meropenem) offer excellent anaerobic and aerobic coverage with meropenem slightly more active against Gram-negatives. Fluoroquinolones (notably levofloxacin, moxifloxacin) have some anaerobic activity and good tissue penetration, but rising resistance limits their role; they are generally reserved for situations such as β-lactam allergy (especially in children) when alternatives are unsuitable [257].

### 7.4. PID Due to Actinomycosis

The risk of infection by *Actinomyces* spp. appears to be increased among IUD users, although the exact risk remains unknown. It is important to note that a positive culture or the presence of *Actinomyces*-like organisms on a Pap test does not necessarily warrant antimicrobial therapy or IUD removal; intervention being recommended only in the presence of clinical evidence of infection [198].

An initial course of intravenous antibiotics for at least two weeks—often extended to 6 weeks—is generally recommended depending on disease severity. Penicillin G remains the recommended IV regimen [258]. IV ampicillin and IV ceftriaxone remain reasonable alternatives [259,260]. In case of penicillin allergy, the selection of alternative agents depends on the type and severity of the allergic reaction. For patients with a non-severe or IgE-mediated allergy, ceftriaxone is generally employed as first line IV therapy [259,261]. However, for patients with severe non-IgE-mediated hypersensitivity reactions doxycycline is preferred, and IV carbapenems such as ertapenem may be considered in consultation with an allergist [262].

Oral regimens include penicillin V or amoxicillin [258,263]. In cases where co-pathogens are suspected amoxicillin-clavulanate is an appropriate option [264]. For penicillin-allergic patients, doxycycline, tetracycline, erythromycin, or azithromycin, have demonstrated in vitro efficacy and have been associated with successful outcomes in limited cases [263,265,266].

The duration of therapy for classic actinomycosis typically ranges from two to six months for mild disease and six to twelve months for severe disease, with treatment continuing for at least one to two months after clinical and/or radiologic resolution of the infection. Longer courses, extending up to 12–18 months, may be required for complicated, invasive infections or in immunocompromised hosts, including those with HIV [267,268].

Shorter durations of therapy for actinomycosis may be considered in select cases, particularly when adequate surgical resection of infected tissue has been performed and bone involvement is absent. Early recognition, rapid clinical response, and smaller, less indurated lesions further support abbreviated therapy. In pelvic actinomycosis associated with IUD use, a reduced treatment course may be sufficient, especially when the IUD is promptly removed and the infection remains localized [269,270,271].

### 7.5. PID Due to Tuberculosis

Management of FGTB follows the same principles as treatment of drug-susceptible pulmonary tuberculosis, in accordance with WHO and CDC/IDSA guidelines [199]. For drug-susceptible cases, the recommended regimen consists of six months of first-line anti-tuberculous therapy: an intensive phase of two months with isoniazid (H), rifampicin (R), pyrazinamide (Z), and ethambutol (E), followed by a four-month continuation phase with isoniazid and rifampicin (2HRZE/4HR) [15].

### 7.6. PID Due to CMV

PID due to CMV is rarely suspected in immunocompetent adults, although a few cases have been reported [156]. However, in immunocompromised populations, including individuals with HIV infection, CMV may cause severe and potentially life-threatening disease. In such cases, early recognition and timely initiation of antiviral therapy are critical [182,272].

For mild to moderate disease, in patients able to tolerate oral therapy, valganciclovir is the preferred treatment. For severe or life-threatening cases, intravenous ganciclovir is recommended, with transition to oral valganciclovir once clinical improvement is achieved. In patients who cannot tolerate first-line therapy, second-line options include maribavir or foscarnet, with the choice guided by disease severity, viral load, and drug related toxicity [273].

### 7.7. PID Due to Parasitic Infection

Infection due to *Enterobius vermicularis* or other parasites should be considered as a potential cause of PID, particularly in cases with unusual presentations or when common bacterial pathogens are not identified [274,165]. Treatment of *Enterobius vermicularis* infection includes anti-helminthic therapy with albendazole, mebendazole, or pyrantel pamoate [275]. Treatment for *Entamoeba* involves systemic therapy with metronidazole for 7–10 days, followed by a luminal agent for 7–20 days depending on the drug. Alternatives to metronidazole include tinidazole, ornidazole, or nitazoxanide [276].

### 7.8. Surgical Management

Although universally accepted guidelines are lacking, certain clinical scenarios may warrant surgical management in PID. Indications include pelvic peritonitis or TOAs, particularly when abscess size exceeds 3–4 cm, or when severe complications such as rupture or septic shock occur. In these cases, prompt initiation of antimicrobial therapy and abscess drainage—preferably via imaging-guided transvaginal approach—should not be delayed. If severe signs are present, surgical intervention, ideally by laparoscopy, may be required, with drainage preferred over excision [277]. In some cases, inflammatory markers CRP and ESR may help guide management and decisions regarding surgical intervention, as elevated levels have been associated with greater disease severity, larger abscess size, and prolonged hospitalization [278,279,280]. Despite factors such as fever, larger abscess size, and higher inflammatory markers being predictive of treatment failure, scoring systems incorporating these parameters have demonstrated poor discriminatory capacity [281].

When surgical intervention is required, laparoscopic surgery appears to offer advantages over open laparotomy. Reported benefits include shorter operation times, reduced need for blood transfusions, and a shorter hospital stay, without increasing the risk of surgical complications, revision surgery, or in-hospital mortality [282].

## 8. Complications and Prognosis

PID is strongly associated with long-term complications, including chronic pelvic pain, infertility, and ectopic pregnancy, and these adverse outcomes may occur not only when treatment is delayed but also despite timely and complete therapy, with affected individuals remaining at risk for recurrent episodes and ongoing morbidity [283,284].

TOA is a severe complication of PID and could lead to morbidity and occasional mortality. Clinical presentation of TOA is similar to that of PID, with the addition of a pelvic mass often noted on examination or imaging [207,285]. TOA’s typical symptoms include fever, abdominal pain, adnexal mass, and abnormal vaginal discharge; thus, obtaining a detailed medical history and performing a thorough clinical examination is mandatory. An auxiliary transabdominal or transvaginal ultrasound may be useful, while in case of diagnostic difficulties, a CT and then an MRI may be helpful [286,287].

A history of PID has been identified as an independent predictor of chronic pelvic pain (CPP) in women [288,289]. CPP occurs in 18–36% of cases and can increase up to 67% in women who experience three or more episodes of PID [2,290]. The underlying mechanisms of CPP are not fully understood, though it is generally thought to result from adhesive disease and damage to the fallopian tubes and ovaries caused by the infection, chronic inflammation due to host immunological responses, or recurrent infections as a consequence of repeated exposures and weakened host defenses [291,292]. Approximately one-third of individuals with PID develop CPP within three years, with more than half experiencing highly intense pain, which is often accompanied by impairments in physical function, overall health, vitality, social engagement, and psychological well-being [290,293]. CPP management may include analgesia, hormonal suppression, and physiotherapy of the pelvic floor, whereas peripheral nerve blocks and sacral nerve neuromodulation may be essential in selected cases.

Individuals with PID might experience hydrosalpinx, a condition resulting from inflammation where the injured fallopian tube may get obstructed by surgery, adhesions, accumulate sterile fluid, and increase in size. The hydrosalpinx persists even after the PID is resolved. In individuals receiving in vitro fertilization (IVF), hydrosalpinx adversely affects pregnancy rates, implantation success, early pregnancy loss, preterm delivery, and live births [294].

Infertility should always be recognized as a potential long-term complication of PID, and women with a history of PID should undergo early assessment when planning pregnancy to enable timely discussion of diagnostic and therapeutic options in case spontaneous conception is not achieved [295]. According to the PID Evaluation and Clinical Health (PEACH) trial, the incidence of infertility among participants with PID was 18% after a three-year follow-up [293]. PID seems to be positively associated with infertility and interestingly, women with a history of PID treatment are considerably more likely to experience infertility compared to women without a history of PID [296]. Potential mechanisms leading to infertility following PID include the upward spread of infection from the cervix to the upper reproductive tract. Disruption of the endocervical mucous plug, mid-cycle uterine peristalsis, and retrograde menstrual flow can facilitate pathogen migration into the pelvis, causing tubal damage and impaired fertility [297].

PID has been associated with an increased risk of adverse pregnancy outcomes, including ectopic pregnancy, with studies showing that women with a history of PID have more than twice the risk compared to those without PID [298,299]. Repeated PID episodes are leading to ongoing injury to fallopian tube and pelvic tissues. Every infection increases tubal adhesions and scarring, resulting in progressively greater infertility rates and ectopic pregnancy [241,300]. The likelihood of ectopic pregnancy rises with the frequency and severity of PID episodes [301]. Of note, non-STI-related PID typically leads to milder peri-tubal adhesions and partial tubal blockages, resulting in a moderate risk of infertility and a relatively weaker association with ectopic pregnancy compared to STI-related PID [301,302].

The association between PID and ovarian cancer remains uncertain. It is indicated that, PID correlates with a heightened risk of ovarian cancer and particularly higher risk in patients with five or more PID episodes [303]. Additionally, there was an increase in borderline ovarian tumors and variations in both serous and non-serous types, however non-STI PID is not associated with ovarian cancer risk, suggesting etiological differences [304,305]. However, if PID independently increases ovarian cancer risk, it can also raise the likelihood of low parity and infertility, which are additional risk factors for ovarian cancer. This is related with evidence suggesting that fallopian tubes, not ovaries, are primarily involved in most cases of related carcinomas [205,207].

Perihepatitis (Fitz-Hugh Curtis Syndrome), which is actually inflammation of the liver capsule and the peritoneal surfaces in the anterior right upper quadrant, is typically associated with STIs such as *N. gonorrhoeae*, *C. trachomatis*, and possibly *M. genitalium*, and is generally not observed in cases of non-STI-related PID [306,307].

Overall, the prognosis of non-STI-related PID is closely linked to early recognition, accurate diagnosis, and timely treatment. Most patients have an excellent outcome when appropriate antimicrobial therapy is initiated promptly, whereas delayed or inadequate management increases the risk of complications such as TOAs, infertility, ectopic pregnancy, chronic pelvic pain, and, less commonly, ovarian malignancy. Ultimately, when the index of suspicion for PID is high, the appropriate diagnostic algorithm is applied, and effective therapy is administered, the long-term prognosis is generally favorable, with preservation of reproductive health and overall quality of life [220].

## 9. Follow-Up and Monitoring

PID is essential to be treated timely and effectively due to the high risk of long-term sequelae [220]. In the absence of strong indications for hospitalization, such as pregnancy, ineffective outpatient treatment, or severe clinical manifestations, mild to moderate PID can be treated on an outpatient basis with a focus on follow-up. More specifically, a clinical reassessment within 48–72 h should be performed, when a substantial improvement in signs and symptoms is expected [215,220,221]. Otherwise, further evaluation should be undertaken with a focus on imaging and repeat clinical assessment, to rule out for potential complications [286].

A high level of suspicion should be maintained for the development of TOAs. Patients with TOAs require IV antibiotic therapy, and significant improvement is expected within 24–48 h, after which a transition to oral antibiotics may be considered. Oral antibiotic therapy continues for at least 14 days. Daily monitoring of the leukocyte count is required to evaluate the process in case of worsening symptoms. In case of no clinical and laboratory improvement, drainage may be necessary. Surgical intervention is needed in case of a ruptured TOA or if the patient is deteriorating [286].

Long-term follow-up is equally important. Infertility may occur due to proximal and/or distal disease of the fallopian tube [295]. When evaluating an individual with a PID history and infertility issues, hysterosalpingography should be performed without delay, and solutions, including surgery, could be assessed [295]. Similarly, high index of suspicion should be raised for ectopic pregnancy or presence of ovarian cancer when evaluating an individual with PID history.

## 10. Prevention

Prevention of non-STI-related PID requires a multifactorial approach. One of the most critical strategies involves the strict application of aseptic techniques during obstetric and gynecological procedures, as post-procedural infection represents a major contributor to PID of non-sexual origin [308]. It is vital for surgical personnel to wear sterile attire, including sterile gloves, gowns, and other protective gear, to avoid contamination of the surgical field. The sterilization of surgical instruments is critical [308]. Minimally invasive surgical procedures must be preferred, if possible, in terms of minimizing the risk of infection as well as minimizing the risk of long-term consequences, such as infertility, attributed to scar tissue formation [308]. Patient education is essential and among others includes post-surgical scrupulous wound care and prompt recognition of symptoms indicating infection, and seeking medical attention [308].

Antibiotic prophylaxis is also important in terms of prevention, though it is not routinely recommended and is tailored to the procedure and the risk factors [32]. In a meta-analysis regarding perioperative antibiotics to avoid infection following a first-trimester abortion, the utilization of preventive antibiotics decreased infection rates after abortion by 41% [32]. The protective impact of antibiotics was evident irrespective of which subgroup was examined including women with a history of PID, women without a documented history of PID and females who received positive results for chlamydial infection during the procedure [309]. For uterine evacuation following abortion or miscarriage, administration of doxycycline or metronidazole, optionally combined with azithromycin in selected high-risk cases, is recommended [310]. Prophylaxis is also indicated for hysterectomy, typically as a single dose of a first- or second-generation cephalosporin, often combined with metronidazole [310]. In contrast, routine antibiotic prophylaxis is not indicated for hysterosalpingography. However, for patients with a prior history of PID, doxycycline or azithromycin are recommended prior to the procedure. Other gynecologic and obstetric interventions, including intrauterine device insertion, endometrial biopsy, and routine hysteroscopy, generally do not require prophylactic antibiotics [310].

Beyond procedural and pharmacological interventions, public health awareness plays a pivotal role in PID prevention. Educational campaigns targeting both the public and healthcare providers are essential, as they increase recognition of symptoms, improve timely access to care, and reduce the risk of complications such as infertility, chronic pelvic pain, or sepsis [220]. A unique reference should be made to the education of special populations, as prompt recognition and effective management are essential [7,311].

While this review addresses PID of non-sexual origin, raising awareness of the sexually transmitted causes remains equally important. Public health education should emphasize safe sexual practices, including the correct and consistent use of barrier contraceptive methods. The United States Preventive Services Task Force (USPSTF) recommends annual screening for *N. gonorrhoeae* and *C. trachomatis* in all sexually active women under 25 years of age, as well as in women aged 25 years and older who possess additional risk factors for infection. [220].

## 11. Future Directions

Even with advancements in non-STI PID studies, significant challenges remain. Studies depend on data that do not have microbiological validation- examination of 11.7 million cases excluding STI/BV testing [16]. Hospital-centric research tends to emphasize severe clinical image, underestimating milder or subclinical cases. Low-income countries fall behind in consistently monitoring non-STI PID, further complicated by diagnostic inequalities (limited laparoscopy/PCR availability). These limitations hinder prevalence and targeted interventions, particularly in areas with low socioeconomic status, where the incidence of non-STDs may be highest. Enhanced monitoring that includes pathogen identification is urgently required for precise epidemiological evaluation [25,312].

A diagnostic gap persists in PID, as existing methods primarily identify only a restricted subset of causative pathogens, thereby failing to comprehensively characterize the broader vaginal microbiota [313,314]. Traditional diagnostic methods, such as microscopy and culture, commonly present low sensitivity and specificity. In contrast, modern approaches, including multiplex PCR and Next-Generation Sequencing, offer enhanced sensitivity, allowing early and precise identification of both sexually transmitted and polymicrobial contributors. On top of that, AI-based approaches can enhance PID management by integrating microbiome profiling to predict disease risk, guide individualized therapy, and facilitate remote monitoring through mobile health platforms [315].

In this context, the vaginal microbiome is crucial for maintaining reproductive health. Dysbiosis, characterized by decreased *Lactobacillus* abundance and a shift toward a more diverse, pathogen-rich microbial community, including *S. aureus*, *K. pneumoniae*, *E. faecalis*, and *E. coli*, has been associated with infertility, particularly in women with PID or idiopathic infertility [316]. Metagenomic analysis of cervical and vaginal microbiota may be useful for detecting changes, such as reduced *Lactobacillus* abundance and increased bacterial diversity [317]. Deep-sequencing technologies may assist in predicting treatment outcomes and recurrence risk, informing strategies to reduce the recurrence rate of BV and consequent PID [318].

The development of noninvasive, biomarker-based diagnostics represents a promising avenue for improving PID detection. Candidate biomarkers include lipopolysaccharides (LPS), and interleukin-6 (IL-6). Both markers are detectable in endometrial tissue, and could serve as effective diagnostic tools, although upscaling would not be easily implemented [319].

## 12. Conclusions

Although originally thought to be primarily a sexually transmitted disease, the rising prevalence of non-STI-related PID highlights the importance of recognizing alternative etiologies. Non-STI-related PID is associated with BV, IUD use, postpartum and post-procedural infections, as well as a diverse array of bacterial, viral, and parasitic pathogens. Currently, no specific clinical guidelines exist for the diagnosis or management of non-STI-related PID, presenting significant challenges for clinicians in tailoring therapy. Empiric treatment frequently relies on extrapolation from STI-based protocols, highlighting the urgent need for evidence-based recommendations that address non-STI etiologies. Given the potential for serious complications such as infertility, chronic pelvic pain, and recurrent infections, future research should prioritize strategies for early diagnosis, accurate identification of causative pathogens, and optimization of therapeutic approaches for non-STI PID.

## Figures and Tables

**Table 1:** Case reports and case series regarding non-STI PID.

Microorganism Identified	Number of Cases	References
Gram positive		
*Actinomyces* spp.	35	[43,44,45,46,47,48,49,50,51,52,53,54,55,56,57,58,59,60,61,62,63,64,65,66,67,68,69,70,71,72,73,74,75,76,77]
*S. pneumoniae*	5	[78,79,80,81,82]
GAS	14	[83,84,85,86,87,88,89,90,91,92,93,94,95,96]
*S. Viridans*	4	[97,98,99,100]
Beta hemolytic *streptococcus* group F	1	[101]
*S. aureus*	3	[102,103,104]
*S. lugdunensis*	1	[105]
*Enterococcus* spp.	2	[106,107]
*Clostridium* spp.	3	[108,109,110]
*Eggerthella lenta*	1	[111]
*Ruminococcus gnavus*	1	[112]
*Abiotrophia/Granulicatella* spp.	1	[113]
*Finegoldia magna*	1	[114]
*Atopobium vaginae*	1	[115]
*Actinobaculum massiliense*	1	[116]
*Nocardia* spp.	2	[117,118]
Gram negative		
*E. coli*	13	[119,120,121,122,123,124,125,126,127,128,129,130,131]
*H. influenzae*	2	[132,133]
*Salmonella* spp.	4	[134,135,136,137]
*K. pneumoniae*	1	[138]
*P. aeruginosa*	1	[139]
*N. meningitidis*	1	[140]
ESBL-Gram-negative pathogen	1	[141]
*Burkholderia pseudomallei*	1	[142]
*B. fragilis*	1	[143]
*Fusobacterium* spp.	4	[144,145,146,147]
*Edwardsiella tarda*	1	[148]
*Leptotrichia* spp.	2	[149,150]
*Citrobacter freundii*	1	[151]
*Pasteurella multocida*	1	[152]
*Porphyromonas asaccharolytica*	1	[100]
*Plesiomonas shigelloides*	1	[153]
*Cambylobacter fetus*	1	[154]
*Chlamydophila abortus*	1	[155]
Viruses		
CMV	5	[156,157,158,159,160]
Fungi		
*Candida* spp.	1	[161]
*Trichosporon beigelii*	1	[162]
Parasites		
Filarian worm-pathogen not identified	1	[163]
*Entamoeba histolytica*	1	[164]
*Enterobius vermicularis*	6	[165,166,167,168,169,170]
*Coccidia* spp.	1	[171]
*Strongyloides stercoralis*	1	[172]
*Plasmodium vivax*	1	[173]
Polymicrobial	12	[24,174,175,176,177,178,179,180,181,182,183,184]
*Mycobacterium* spp.	15	[185,186,187,188,189,190,191,192,193,194,195,196]

Abbrevations: *E. coli*: *Escherichia coli*; *S. pneumoniae*: *Streptococcus pneumoniae*; *S. viridans*: *viridans group streptococci*; *S. aureus*: *Staphylococcus aureus*; *S. lugdunensis*: *Staphylococcus lugdunensis*; *B. fragilis*: *Bacteroides fragilis*; *H. influenzae*: *Haemophilus influenzae*; *K. pneumoniae*: *Klebsiella pneumoniae*; *P. aeruginosa*: *Pseudomonas aeruginosa*; *N. meningitidis*: *Neisseria meningitidis*; CMV: cytomegalovirus; GAS: Group A *Streptococcus*; ESBL: extended-spectrum β-lactamase.

**Table 2:** Regimens for treating non-STI PID.

Empiric Treatment (Hospitalized Individuals)		
Regimen	Dosage	Notes
Ceftriaxone or cefoxitin or cefotetan + Doxycycline + Metronidazole	Ceftriaxone 1 g IV q24h/Cefoxitin 2 g IV q6h/Cefotetan 2 g IV q12h plus Doxycycline 100 mg orally or IV q12h plus Metronidazole 500 mg orally or IV q12h	Oral doxycycline is preferred if tolerated due to equivalent bioavailability.
Alternative regimens		
Clindamycin + Gentamicin	Clindamycin 900 mg IV q8h plus Gentamicin 3–5 mg/kg IV daily or 2 mg/kg loading dose then 1.5 mg/kg q8h	
Ampicillin–sulbactam + Doxycycline	Ampicillin–sulbactam 3 g IV q6h plus Doxycycline 100 mg q12h	
Ampicillin + Clindamycin + Gentamicin	Ampicillin 2 g IV q6h plus Clindamycin 900 mg IV q8h plus Gentamicin 2 mg/kg loading dose then 1.5 mg/kg q8h	
Imipenem-cilastatin	Imipenem-cilastatin 500 mg IV q6h	
Azithromycin or moxifloxacin or levofloxacin + Metronidazole	Azithromycin 500 mg IV daily for 1–2 days, then 250 mg orally daily for 7 days or moxifloxacin 400 mg orally once daily or levofloxacin 500 mg IV once daily plus Metronidazole 500 mg orally q12h	Alternative if cephalosporin allergy is present.
**Empiric Treatment (Non-Hospitalized individuals)**		
Ceftriaxone or Cefoxitin or Cefotaxime, or Ceftizoxime + Doxycycline or azithromycin + Metronidazole	Ceftriaxone 500 mg IM (<150 kg) or 1 g IM (≥150 kg), Cefoxitin 2 g IM with Probenecid 1 g orally, Cefotaxime 1 g IM, or Ceftizoxime 1 g IM + Doxycycline 100 mg orally q12h or Azithromycin 500 mg daily for 1–2 days, then 250 mg daily (or 1 g once weekly for 2 weeks) plus Metronidazole 500 mg orally q12h	For patients who are intolerant to doxycycline, azithromycin is preferred.
Doxycycline + Clindamycin	Doxycycline 100 mg q12h plus Clindamycin 450 mg q6h	For patients who are intolerant to metronidazole.
Levofloxacin or Moxifloxacin + Metronidazole	Levofloxacin 500 mg qd or Moxifloxacin 400 mg orally qd plus Metronidazole 500 mg q12h	Alternative if cephalosporin allergy is present.
Targeted Therapy (for specific pathogens)		
***Enterobacterale*** **s and ** ***P. aeruginosa***		
Ceftriaxone or cefepime or ceftazidime	Ceftriaxone 2 g IV qd or Ceftazidime 2 g IV q8h or Cefepime 2 g IV q8h	Ceftazidime and Cefipime provide coverage for *P. aeruginosa.*
Aztreonam or Ciprofloxacin or Levofloxacin	Aztreonam 2 g every q8h or Ciprofloxacin 400 mg IV q12h or Levofloxacin 750 mg IV qd	For severe b-lactam allergy.
Ertapenem or Piperacillin-tazobactam or Meropenem or Imipenem	Ertapenem 1 g IV qd or Piperacillin-tazobactam 4.5 g q6h or Meropenem 2 g q8h or Imipenem 500 mg IV q6h	Alternative regimens. Ertapenem does not provide coverage for *P. aeruginosa.*
Ceftolozane-tazobactam	Ceftolozane-tazobactam 2.5 g q8h	Suitable for ESBL-producing pathogens.
Ceftazidime-Avibactam or Imipenem-Cilastatin-Relebactam or Meropenem-Vaborbactam or Cefiderocol	Ceftazidime-Avibactam 2.5 g q8h IV or Imipenem-Cilastatin-Relebactam 1.25 g q6h IV or Meropenem-Vaborbactam 4 g q8h IV or Cefiderocol 2g q8h IV	Reserved for CRE, CRPA or CRAB.
***Anaerobes***		
Metronidazole	Metronidazole 500 mg q12h	First-choice regimen.
Clindamycin or Amoxicillin–clavulanate or Τicarcillin–clavulanate or Piperacillin–tazobactam or Ampicillin–sulbactam or Meropenem or Imipenem	Clindamycin 450 mg q6h or Amoxicillin–clavulanate 1 g q8h or Τicarcillin–clavulanate 3.1g q6h or Piperacillin–tazobactam 4.5 g q6h or Ampicillin–sulbactam 3 g q6h or Meropenem 2 g q8h or Imipenem 500 mg IV q6h	Alternative regimen.
**PID due to Actinomycosis**		
Penicillin G IV or Ampicillin IV followed by Penicillin V or Amoxicillin per os	Penicillin G 20–24 million units daily, divided q4–6h or Ampicillin IV 2 g q4–6h followed by Penicillin V or Amoxicillin per os 2–3 g per day, divided in 3–4 doses	
	2 g q4–6h	Alternative regimen.
Ceftriaxone IV	2 g qd	For non-severe or IgE-mediated allergy.
Doxycycline IV	100 mg q12h	Preferred for severe non-IgE-mediated allergy.
Carbapenems (e.g., Ertapenem)	1 g IV every 24 h	For severe allergy.
Doxycycline or Tetracycline or Erythromycin or Azithromycin per os	Doxycycline 100 mg q12h or Tetracycline 500 mg q6h or Erythromycin 500 mg q6h or Azithromycin 500 mg qd	For penicillin-allergic patients.
**PID due to CMV**		
Valganciclovir per os	900 mg q12h	Mild–moderate CMV disease.
Ganciclovir IV	5 mg/kg q12h	Severe/life-threatening CMV disease.
Maribavir or foscarnet	Dose individualized	Alternative regimen.
**PID due to parasitic infection**		
*Enterobius vermicularis*		
Albendazole	400 mg single dose repeated after 2 weeks	
Mebendazole or	Mebendazole 100 mg single dose, repeated after 2 weeks or Pyrantel pamoate 11 mg/kg (max 1 g) single dose, repeated after 2 weeks	Alternative option..
*Entamoeba histolytica*		
Metronidazole + Luminal Agent (Paromomycin or Diiodohydroxyquin or Diloxanide furoate)	Metronidazole 500–750 mg orally q8h plus Paromomycin: 25–30 mg/kg/day divided q8h Diiodohydroxyquin: 650 mg orally Diloxanide furoate: 500 mg orally q8h	Alternatives to metronidazole include tinidazole, ornidazole, nitazoxanide
**PID due to Tuberculosis**		
Isoniazid + Rifampicin + Pyrazinamide + Ethambutol followed by two drug regimen (Isoniazid+ Rifampicin) next 8 weeks	Isoniazid 300 mg daily plus Rifampicin 10 mg/kg (8–12 mg) once daily plus Pyrazinamide 1000 mg (40–<55 kg), 1500 mg (55–75 kg), 2000 mg (>75 kg) daily plus Ethambutol 15 mg/kg orally once a day for initial treatment or Initial dose: 25 mg/kg orally once a day for 60 days Maintenance dose: 15 mg/kg orally once a day for previous antituberculosis treatment	(First 8 weeks)

IV: Intravenous; qd: Every 24 h; q12h: Every 12 h; q8h:Every 8 h; q6h:Every 6 h; STI: Sexually Transmitted Infection; PID: Pelvic Inflammatory Disease, IV; intravenous, IM; intramuscular, *P. aeruginosa*; *Pseudomonas aeruginosa*, MDR; multidrug-resistant, MBL; metallo-β-lactamase, CMV; cytomegalovirus, IgE; immunoglobulin E, e.g., for example, per os; orally, CRE: Carbapenem resistant *Enterobacteriales*; CRPA: Carbapenem resistant *Pseudomonas aeruginosa*; CRAB: Carbapenem Resistant *Acinetobacter baumanii*; ESBL: Extended Spectrum β lactamase.

## Supplementary Materials

The following supporting information can be downloaded at: https://www.mdpi.com/article/10.3390/microorganisms13122813/s1, Figure S1: PRISMA diagram.

## Abbreviations

The following abbreviations are used in this manuscript:

AI	Artificial Intelligence
AIDS	Acquired Immunodeficiency Syndrome
BL/BLI	Beta-lactam/Beta-lactamase inhibitor
BV	Bacterial Vaginosis
CA-125	Cancer Antigen 125
CDC	Centers for Disease Control and Prevention
CMV	Cytomegalovirus
CPP	Chronic Pelvic Pain
CRP	C-reactive Protein
CRAB	Carbapenem Resistant *Acinetobacter baumanii*
CRE	Carbapenem resistant *Enterobacteriales*
CRPA	Carbapenem resistant *Pseudomonas aeruginosa*
CT	Computed Tomography
DNA	Deoxyribonucleic Acid
*E. coli*	*Escherichia coli*
ESBL	Extended-Spectrum β-Lactamase
ESR	Erythrocyte Sedimentation Rate
FGTB	Female Genital Tract Tuberculosis
GI	Gastrointestinal
GAS	Group A *Streptococcus*
*H. influenzae*	*Haemophilus influenzae*
HSG	Hysterosalpingography
HIV	Human Immunodeficiency Virus
HR	Rifampicin + Isoniazid regimen
HRZE	Isoniazid, Rifampicin, Pyrazinamide, and Ethambutol regimen
IBD	Inflammatory Bowel Disease
IDSA	Infectious Diseases Society of America
IFN-γ	Interferon Gamma
IgA	Immunoglobulin A
IgE	Immunoglobulin E
IL-1	Interleukin-1
IL-6	Interleukin-6
IL-8	Interleukin-8
IM	Intramuscular
IUD	Intrauterine Device
IV	Intravenous
*K. pneumoniae*	*Klebsiella pneumoniae*
LPS	Lipopolysaccharide
MBL	Metallo-β-lactamase
*M. genitalium*	*Mycoplasma genitalium*
*M. hominis*	*Mycoplasma hominis*
*M. tuberculosis*	*Mycobacterium tuberculosis*
MDR	Multidrug-Resistant
MMP-9	Matrix Metalloproteinase-9
MRI	Magnetic Resonance Imaging
NAAT	Nucleic Acid Amplification Test
NF-κB	Nuclear Factor Kappa-Light-Chain-Enhancer of Activated B Cells
NLR	Neutrophil-to-Lymphocyte Ratio
*N. gonorrhoeae*	*Neisseria gonorrhoeae*
*N. meningitidis*	*Neisseria meningitidis*
*P. aeruginosa*	*Pseudomonas aeruginosa*
PCR	Polymerase Chain Reaction
PID	Pelvic Inflammatory Disease
PCT	Procalcitonin
RNA	Ribonucleic Acid
*S. aureus*	*Staphylococcus aureus*
*S. lugdunensis*	*Staphylococcus lugdunensis*
*S. pneumoniae*	*Streptococcus pneumoniae*
*S. viridans*	Viridans Group *Streptococci*
STD	Sexually Transmitted Disease
STI	Sexually Transmitted Infection
TB	Tuberculosis
TGF-β	Transforming Growth Factor Beta
TLR	Toll-Like Receptor
TOA	Tubo-Ovarian Abscess
USPSTF	United States Preventive Services Task Force
WHO	World Health Organization
WBC	White Blood Cell
