# Supplementary material for: Non-Sexually Transmitted Infection (STI)-Related Pelvic Inflammatory Disease (PID)
Source: Microorganisms. 2025 Dec 10;13(12):2813. doi: 10.3390/microorganisms13122813 (PMC12735436; doi:10.3390/microorganisms13122813)
Supplement: Supplementary file 1 [file microorganisms-13-02813-s001.zip › microorganisms-3979033-supplementary.pdf]

A literature search was conducted by three independent reviewers over a 30-year period (1995–2025) to identify cases or case series of individuals with PID or TOAs associated with non-sexually transmitted organisms. The search was performed in PubMed using the following MeSH terms: pelvic inflammatory disease, tubo-ovarian abscess, endometritis, non-sexually transmitted infections, Actinomyces, Mycobacterium tuberculosis, non-sexually active, oophoritis, salpingitis, Escherichia coli, anaerobes. The identification, screening, and inclusion of reports were conducted according to the PRISMA framework, as depicted in Sup. Figure S1.

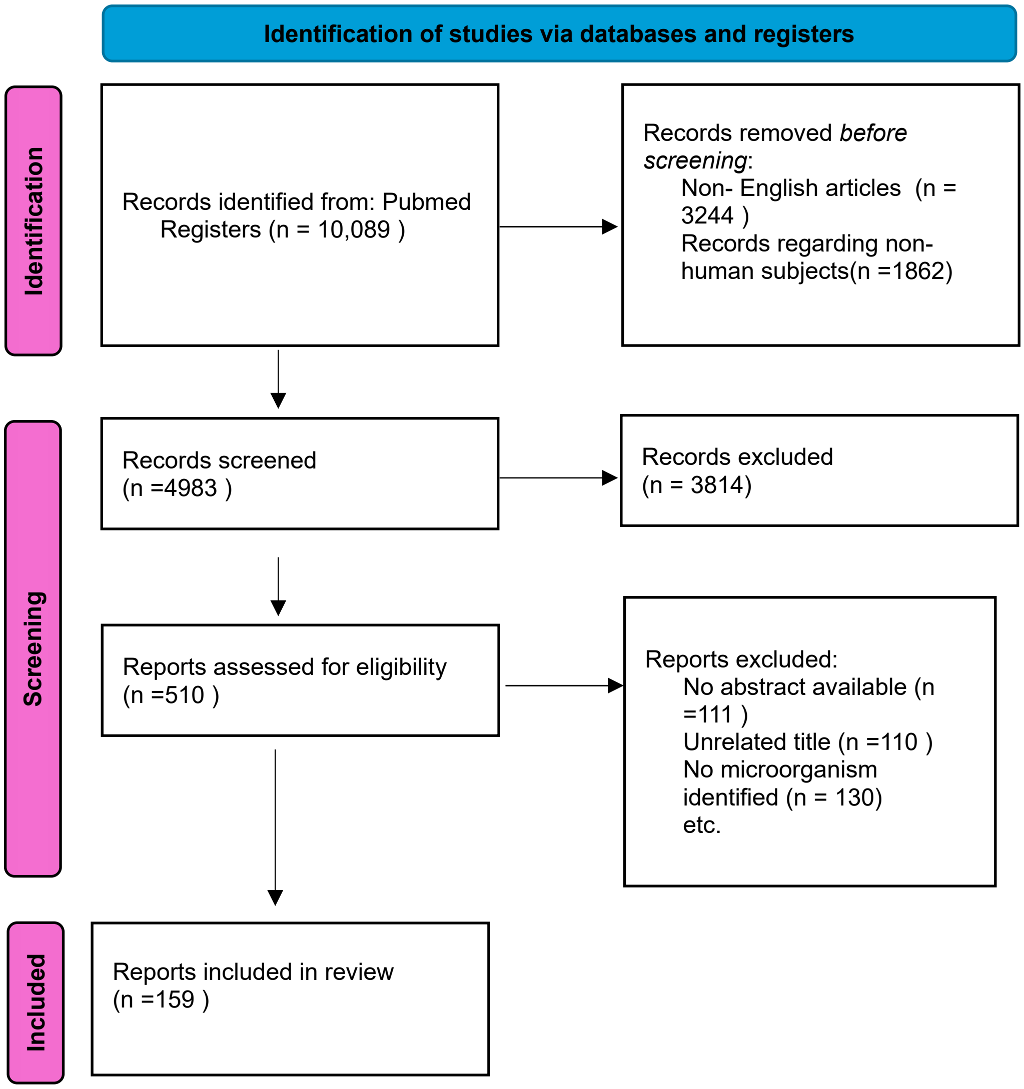

Figure S1. PRISMA diagram.

Articles that were not published in English or did not involve human subjects were excluded from screening to ensure relevance and applicability to clinical practice; an additional 3,814 articles did not meet the specific criteria of our search, resulting in 510 reports that were assessed for eligibility. From the 510 reports, studies were further excluded for the following reasons: no abstract available (n = 111), unrelated title (n = 110), no microorganism identified (n = 131), among

others, resulting in the final set of 158 reports, case series and cohort studies included in the review.
